# Stochastic models for the in silico simulation of synaptic processes

**DOI:** 10.1186/1471-2105-9-S4-S7

**Published:** 2008-04-25

**Authors:** Andrea Bracciali, Marcello Brunelli, Enrico Cataldo, Pierpaolo Degano

**Affiliations:** 1 Dipartimento di Informatica, Università di Pisa, Pisa I-56127, Italy; 2 Dipartimento di Biologia, Università di Pisa, Pisa I-56127, Italy

## Abstract

**Background:**

Research in life sciences is benefiting from a large availability of formal description techniques and analysis methodologies. These allow both the phenomena investigated to be precisely modeled and virtual experiments to be performed *in silico*. Such experiments may result in easier, faster, and satisfying approximations of their *in vitro/vivo* counterparts. A promising approach is represented by the study of biological phenomena as a collection of interactive entities through process calculi equipped with stochastic semantics. These exploit formal grounds developed in the theory of concurrency in computer science, account for the not continuous, nor discrete, nature of many phenomena, enjoy nice compositional properties and allow for simulations that have been demonstrated to be coherent with data in literature.

**Results:**

Motivated by the need to address some aspects of the functioning of neural synapses, we have developed one such model for synaptic processes in the *calyx of Held*, which is a glutamatergic synapse in the auditory pathway of the mammalia. We have developed such a stochastic model starting from existing kinetic models based on ODEs of some sub-components of the synapse, integrating other data from literature and making some assumptions about non-fully understood processes. Experiments have confirmed the coherence of our model with known biological data, also validating the assumptions made. Our model overcomes some limitations of the kinetic ones and, to our knowledge, represents the first model of synaptic processes based on process calculi. The compositionality of the approach has permitted us to independently focus on tuning the models of the pre- and post- synaptic traits, and then to naturally connect them, by dealing with “interface” issues. Furthermore, we have improved the expressiveness of the model, e.g. by embedding easy control of element concentration time courses. Sensitivity analysis over several parameters of the model has provided results that may help clarify the dynamics of synaptic transmission, while experiments with the model of the complete synapse seem worth explaining short-term plasticity mechanisms.

**Conclusions:**

Specific presynaptic and postsynaptic mechanisms can be further analysed under various conditions, for instance by studying the presynaptic behaviour under repeated activations. The level of details of the description can be refined, for instance by further specifying the neurotransmitter generation and release steps. Taking advantage of the compositionality of the approach, an enhanced model could then be composed with other neural models, designed within the same framework, in order to obtain a more detailed and comprehensive model. In the long term, we are interested, in particular, in addressing models of synaptic plasticity, i.e. activity dependent mechanisms, which are the bases of memory and learning processes.

More on the computer science side, we plan to follow some directions to improve the underlying computational model and the linguistic primitives it provides as suggested by the experiments carried out, e.g. by introducing a suitable notion of (spatial) locality.

## Background

During the last decades, the development of high-throughput technologies has produced a huge amount of information in the field of neurobiology, requiring the utilization of mathematical modeling to describe the complex dynamics of biological processes and stimulating new collaborations among biologists, physicists and computer scientists [1,2]. The building blocks of neural systems are the neurons, which are specialized eukaryotic biological cells able to communicate with each other at highly specialized contact sites, called synapses. In general, each neuron consists of a somatic cellular body, on which a variable number of thin elongated structures, called dendrites, converge and from which a long single structure, called axon, emerges, branching in several synaptic terminals. The synaptic terminals of the transmitting neuron (the presynaptic element) send signals by releasing chemical molecules (neurotransmitters) to the dendritic, somatic or axonic, part of the receiving neuron (postsynaptic term) [3].

The synapses are the places of functional contacts between neurons, where the information is stored and transmitted from one to another neuron. Synaptic transmission is a complex process not completely understood: “Our current knowledge concerning synaptic transmission in neuronal networks of the brain is comparable to a puzzle in which most of the pieces are still missing” [4]. Current knowledge on synapses is based on the analysis of a limited number of experimental synaptic models, which were chosen for their experimental accessibility.

**Figure 1:**
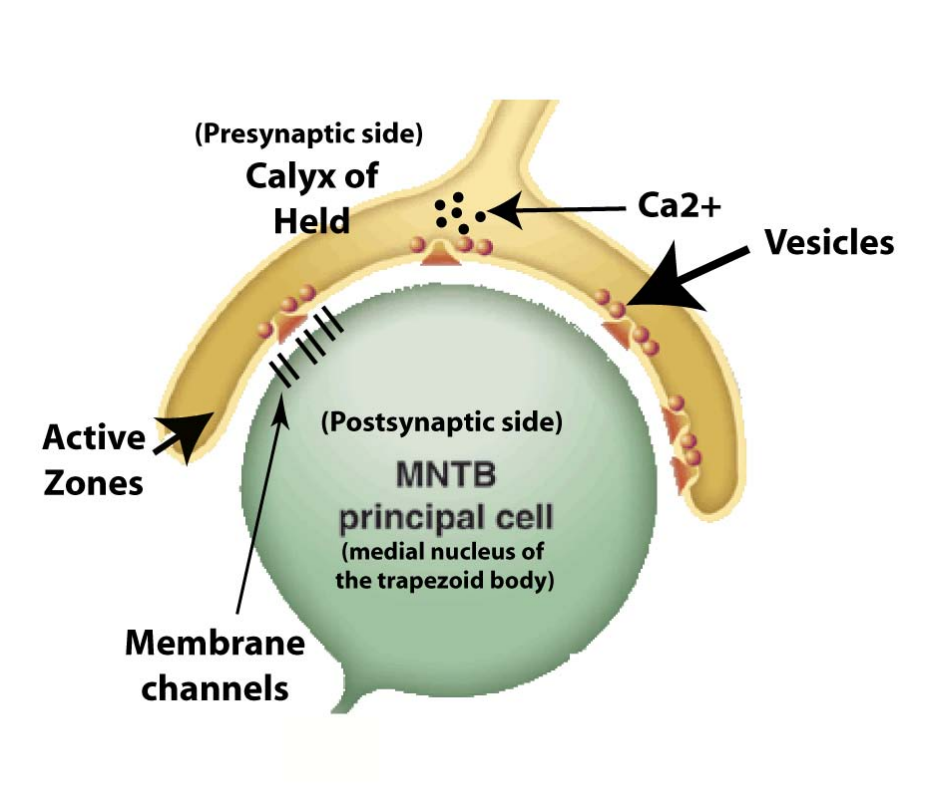
**A picture of the Calyx of Held**. The *calyx of Held* is an excitatory glutamatergic synapse in the auditory brainstem circuit. It is part of the relay pathway subserving sound-source localization. It synapses onto a given single MNTB (medial nucleus of the trapezoid body) principal cell. A gradient of *Ca*^2+^ ions in the presynaptic trait activates vesicles – five ions bind a vesicle. Activated vesicles release neurotransmitters in the synaptic cleft. Membrane channels in the postsynaptic trait react to neurotransmitters.

On the presynaptic side (sketched in Figure 1), one of the issues of the transmission concerns the involvement of calcium ions in the control of neurotransmitter release, which was hypothesized many years ago and more recently demonstrated [5]. The neurotransmitter release is mediated by exocitosis of synaptic vesicles (small elements containing the neurotransmitters) located at the presynaptic so-called active zones [6]. The electrical signals (action potentials) arriving at the synaptic terminal induce the opening of the *Ca*^2+^ channels. The transient elevation of the internal *Ca*^2+^ concentration in the presynaptic terminal triggers synaptic vesicle exocitosis, and hence the neurotransmitter release (calcium-triggered-release hypothesis). Interestingly, chemical messengers (intracellular) and modulators (extracellular) regulate the relationship between action potential and release in a synaptic terminal, which is also altered by the repeated activity. All these things make the presynaptic terminal a kind of computational unit, which changes its output based on its previous activity and ongoing modulation.

It must be noted that the *Ca*^2+^ signals were unaccessible to direct measurements up to few years ago. Theoretical and functional studies have suggested that calcium acts on presynaptic vesicles by a local, huge and short-lived elevation of its concentration. The locality and rapidity of the concentration variation render the study of this phenomenon not approachable with the conventional microscopic imaging techniques. Among the methods envisaged to overcome these limitations, one very fruitful approach is the so-called reverse approach, in which *Ca*^2+^ uncaging is induced in the presynaptic element. The uncaging method induces spatially homogeneous *Ca*^2+^ elevation, implying that measuring the *Ca*^2+^ fluorescent indicator gives an indication of the real *Ca*^2+^ sensed by the vesicles. This experimental method has been applied to the study of the large synapse of the auditory tract of the central nervous system, called *calyx of Held*. Moreover, it has been possible to build a minimal kinetic model for the process of the *Ca*^2+^ triggered vesicle release. Also, one can infer local *Ca*^2+^ signal waveform which is compatible with the experimental data on the time course and amplitude of release [7,8].

Most of the models treating the issues of the calcium triggered release present some methodological limitations. These models, and among them the *calyx of Held* model, use differential equations to describe the time course of [*Ca*^2+^], the *Ca*^2+^ concentration (*mole* × *liter*^−1^) interacting with the synaptic vesicles. This approach implies that [*Ca*^2+^] is continuous, while it is clearly not [9]. For example, a cubic volume with edge length 60 *nm* and a *Ca*^2+^ concentration of 10 *μ*M contains about a single free ion. Another common assumption is that the binding of the *Ca*^2+^ to the release sensors of the vesicle does not affect the [*Ca*^2+^] concentration [9]. Also this assumption is not exactly adequate: considering that the dimensions of the vesicle diameters range in the interval 17-45 *nm*, an average interaction volume, a cube with edge length 60 *nm* say, would contain few *Ca*^2+^ ions, and when some of them bind to the vesicle sensors, the number of calcium ions could change substantially.

In general, when the fluctuations in the molecular population levels are relevant, e.g. when the numbers per unit volume of the molecular species involved are small, the stochastic approach has to be preferred. The fluctuations, that can be seen as random variations about a mean number of molecules, can significantly alter the dynamics of biochemical pathways. Just to cite few examples, fluctuations might decrease or increase the steepness of a nonlinear stimulus-response relationship. In other cases, via the so-called stochastic resonance, they can increase the reliability of response to small signals [10].

Hence, in these cases, the use of a stochastic approach appears to be much more appropriate, since the deterministic approach fails to capture the nature of chemical kinetics, which at low concentrations is discrete and stochastic [11]. In the stochastic approach, the system is described by the so-called “master equation”, which is usually intractable. A stochastic simulation algorithm has originally been proposed in [12] to overcome this difficulty.

On the postsynaptic side (sketched in Figure 1), the signal is transmitted when the neurotransmitters and the membrane receptors interact. The synaptic function is determined by many factors, such as the the speed of clearance of the neurotransmitters from the cleft, which determines the amount of postsynaptic receptors that bind the transmitters. This mechanism is also still poorly understood, because these events are beyond the power of experimental observations [13]. Due to its considerable size, the *calyx of Held* synapse represents a useful model system for studying the postsynaptic element, too. For this reason it has been the object of several experimental and theoretical works, e.g. [14,15]. In general, the description of the postsynaptic interaction is made through deterministic approaches, which mainly rely on the use of molar concentration for the quantitative measure of the neurotransmitters [14,16]. However, these suffer from the same limitations discussed for the case of the presynaptic vesicle release, and make the use of stochastic models preferable.

It is worth noting that the approach to devise models of the whole synaptic function has been mainly based so far on deterministic ODE descriptions [16] and the utilization of a stochastic approach, typically based on [12], is just at the beginning [17].

Recently, stochastic techniques have also been adopted in computer science to model quantitative aspects of interactive systems within concurrency theory. The aim of concurrency theory is to model the behaviour and the structure of systems composed of autonomous computational entities, which dynamically interact with one another, possibly reconfiguring the system itself. At the beginning, stochastic models have been used to study performance/time related properties, e.g. [18]. The strong analogies between concurrent and living systems, summarised in “cells as computation” [19], are contributing to the development of Systems Biology [20,21], a systemic approach to living system modeling. According to this metaphor, cells, molecules and biological “active” components, i.e. those capable of exhibiting a behaviour, are assimilated to computer processes, the computational units of a concurrent software system. By communicating, processes may exchange information or synchronise themselves. Then, biological interaction corresponds to process communication. A biological experiment, or biological activity in general, has then a direct correspondence into a computation. That is, biological processes can not only be simulated by *in silico* experiments, but also it is possible to formally reason about their computational models and infer properties of interest. *Process calculi* are a formalism to describe such models: systems are *compositionally* described in terms of suitable *abstractions* of their component behaviour. Several process calculi whose “operators” are oriented to describing different aspects of biological interaction have been proposed, e.g. [22-25]. Some of these calculi have been equipped with a stochastic semantics in order to study the quantitative dynamics of systems, e.g. [23,26-28]. This approach benefits from conjugating the abstract and compositional algebraic models, the possibility of precisely describing their semantics and formally reasoning about them, and the quantitative analysis provided by stochastic semantics. Executable implementations of the calculi and analysis tools are provided.

In this context, motivated by addressing some aspects of the functioning of neural synapses, we have first developed a stochastic model of the calcium triggered release in the *calyx of Held* synapse. Our work starts from a deterministic model presented in [7], from which we have derived a suitable stochastic model. This has subsequently been formalised in terms of Pi-calculus [29], a process calculus designed for concurrency and mobility. The behaviour of *Ca*^2+^ and vesicles has been described and composed to form the presynaptic terminal. Then, by the same approach, we have developed a stochastic model of the postsynaptic receptor kinetics, starting from the semi-deterministic model presented in [14,16].

Finally, we have obtained a whole model of signal transmission throughout the synapse by suitably composing the two models of the pre- and post- synaptic sides developed individually. It must be noted that our model represents a fragment of the whole synapse focusing on a cluster of functionally related active zones. However, due to the morpho-functional organization of this synapse consisting of similar, independent active zones functioning in parallel, the behavior of the whole synapse can be easily obtained by scaling operation.

Model development has benefited from the above mentioned features, like modular design, abstract representation of the component functioning and stochastic interpretation of the system dynamics. Modular and abstract design, in particular, has been revealed to be suitable to facilitate the incremental development of a more complete and detailed model.

*In silico* experiments have been carried out by means of the stochastic Pi-calculus simulator SPiM [30], which represents one of the most complete and expressive simulation environments for stochastic calculi currently available. Obtained results are coherent with those from the deterministic models and with others in literature. The expressiveness of the model has allowed us to easily describe environmental conditions of the experiments, such as modulating presynaptic calcium waves. This has helped us to identify some critical points of the model and the parts of the overall process that seem to play a more relevant role. Beyond the experiments here reported, our approach appears to be interesting in the long term for the development of more comprehensive stochastic models of synaptic functioning. Moreover, the experiments carried out so far have been given interesting insights about the linguistic mechanisms that could usefully extend the expressiveness of the calculus.

The model is described in the next section, where we also report on some of the experiments done. The computational implementation of the model is reported in the last section *Methods*. Perspectives of the approach are discussed in *Conclusions*.

## Results and Discussion

We have applied a stochastic approach, based on the algorithm introduced in [12], to describe the presynaptic calcium triggered release mechanisms and the postsynaptic channel opening, studied in the model system of the synapse *calyx of Held*. For the presynaptic terminal we have modeled both the cases of step-like and wave-like calcium uncaging, while we have considered membrane receptor activity for the postsynaptic side.

### Step-like calcium uncaging

Our starting point was the following phenomenological kinetic model, described in [7]. (Slightly imprecisely, hereafter we shall use the notation $A+B\begin{array}{c} \overset{to}{\to }\\ \underset{back}{\leftarrow } \end{array}C+D\begin{array}{c} \overset{\ldots }{\to }\\ \underset{\ldots }{\leftarrow } \end{array}\ldots$ to represent the reversible reaction from *A* and *B* to *C* and back, and then, the produced *C* is involved, together with *D*, in the next (reversible) reaction producing …. I.e., the formula does not intend to represent neither *A* + *B* ← *C* + *D* nor *A* + *B* → *C* + *D*). Vesicles are activated through a process of five calcium binding steps and eventually released with a given kinetic rate constant. This process is determined by a cooperativity factor *b*. Vesicles are represented as *V*, the intracellular calcium as $Ca_{i}^{2+}$, the released vesicles as *T* and the kinetic rate constant relative to vesicle release is γ = 6000 *s*^−1^. The values of the other constants of the model are *k_on_* = 9 × 10^7^*M*^−l^*s*^−l^, *k_off_* = 9500 *s*^−1^ and *b* = 0.25:

$$\begin{array}{l} V+Ca_{i}^{2+}\begin{array}{c} \overset{5k_{on}}{\to }\\ \underset{k_{off}b^{0}}{\leftarrow } \end{array}V_{Ca_{i}^{2+}}+Ca_{i}^{2+}\begin{array}{c} \overset{4k_{on}}{\to }\\ \underset{2k_{off}b^{1}}{\leftarrow } \end{array}V_{2Ca_{i}^{2+}}+Ca_{i}^{2+}\begin{array}{c} \overset{3k_{on}}{\to }\\ \underset{3k_{off}b^{2}}{\leftarrow } \end{array}V_{3Ca_{i}^{2+}}\\ V_{3Ca_{i}^{2+}}+Ca_{i}^{2+}\begin{array}{c} \overset{2k_{on}}{\to }\\ \underset{4k_{off}b^{3}}{\leftarrow } \end{array}V_{4Ca_{i}^{2+}}+Ca_{i}^{2+}\begin{array}{c} \overset{k_{on}}{\to }\\ \underset{5k_{off}b^{4}}{\leftarrow } \end{array}V_{5Ca_{i}^{2+}}\overset{\gamma }{\to }T \end{array}$$

The parameters of the kinetic model were computed by a fitting of the experimental data. These had been obtained by elevating the intracellular presynaptic [*Ca*^2+^] in a controlled, homegeneous and step-like manner [7].

The previous equations have been transformed by utilising the relationship between the stochastic rate constants (*c*) and deterministic rate constants (*k*) [31]. For reactions of the first order, *c* = *k*. For reactions of second order, the relationship becomes *c* = *k*/(*NA* × *Vol*), where *NA* represents the Avogadro's number and *Vol* the volume of the reaction. Hence, in order to determine the values of the stochastic rate constants, we need to estimate the value of *Vol*. Spatially, the calyx of Held is organized as a “parallel” arrangement of a large array of active zones, ranging from 300 to almost 700 [32]. Each active zone contains up to 10 vesicles and they are clustered in groups of about 10 of them, in a volume having a diameter of almost 1 μm. Each action potential activates all the active zones. Such particular morpho-functional organization of this synapse has allowed us to model a subunit of the presynaptic element, consisting of a cluster of 10 active zone, each containing 10 vesicles, in a volume of 0.5 10^−15^*liter*. With this volume estimate, we have obtained the following values for the stochastic constants: *b* = 0.25, *c_on_* = 9 × 10^7^ / (6.02 × 10^23^ × 0.5 × 10^−15^) *s^−l^* = 0.3 *s*^−l^, *c_off_* = 9500 *s*^−l^, γ = 6000 *s*^−l^, and the following numbers of *Ca*^2+^ ions: 300, 3000 and 6000, corresponding to molar concentrations [*Ca*^2+^] of 1, 10 and 20 *μ*M. The equations of the stochastic model follow:

$$\begin{array}{l} V+Ca_{i}^{2+}\begin{array}{c} \overset{5c_{on}}{\to }\\ \underset{c_{off}b^{0}}{\leftarrow } \end{array}V_{Ca_{i}^{2+}}+Ca_{i}^{2+}\begin{array}{c} \overset{4c_{on}}{\to }\\ \underset{2c_{off}b^{1}}{\leftarrow } \end{array}V_{2Ca_{i}^{2+}}+Ca_{i}^{2+}\begin{array}{c} \overset{3c_{on}}{\to }\\ \underset{3c_{off}b^{2}}{\leftarrow } \end{array}V_{3Ca_{i}^{2+}}+Ca_{i}^{2+}\\ \;\;V_{3Ca_{i}^{2+}}+Ca_{i}^{2+}\begin{array}{c} \overset{2c_{on}}{\to }\\ \underset{4c_{off}b^{3}}{\leftarrow } \end{array}V_{4Ca_{i}^{2+}}+Ca_{i}^{2+}\begin{array}{c} \overset{2c_{on}}{\to }\\ \underset{4c_{off}b^{4}}{\leftarrow } \end{array}V_{5Ca_{i}^{2+}}\overset{\gamma }{\to }T \end{array}$$

Note that, according to [7], we represent the fusion of each vesicle as the production of one released vesicle *T*, abstracting, for simplicity, from the actual quantities of neurotransmitters involved in the final step of the release process.

**Figure 2:**
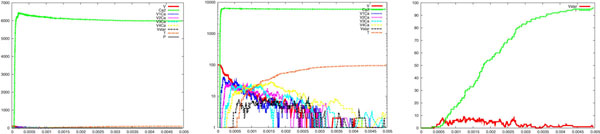
**Step-like calcium uncaging.** Simulation results showing vesicle release following a step-like *Ca*^2+^ uncaging. From left to right are reported the time course of *Ca*^2+^, the logarithmic scale of it, showing the intermediate states of calcium binding, and a focus on vesicle activation (*V star*) and the cumulative amount of vesicle released (*T*). It can be observed that the pool of vesicles is almost completely depleted within 3 *ms*. The following set of parameters were utilised: V=100; *Ca*^2+^=6000; *C_on_*=0.3; *C_off_*=9500; γ=6000; b=0.25.

We have performed a series of simulations which confirmed the results obtained with the deterministic model: high sensitivity of vesicles to calcium concentrations [7]. Moreover, it is known that the local calcium concentration can be much lower than 100 *μ*M in the calyx of Held [7], while several other synapses require a calcium concentration in the range of 100-300 *μ*M for triggering vesicle releases [6]. Our results also confirm this fact, by showing that concentrations as low as 1, 10 and 20 *μ*M are able to deplete the releasable pool in a few milliseconds. In the following we will report a sample of our results. Each one consists of three pictures reporting the time course of *Ca*^2+^, the logarithmic scale of it, which shows intermediate states of calcium binding, and a focus on vesicle activation (**Vstar**) and release (T), respectively. Figure 2 shows simulation results for the following parameters: *V* = 100; *Ca*^2+^ = 6000; *c_on_* = 0.3; *c_off_* = 9500; γ = 6000; *b* = 0.25. It can be observed that the pool of vesicles is 80% depleted within 3 *ms*, coherently to the experimental findings [7].

### Wave-like calcium uncaging

The technique of step-like calcium uncaging overcomes the limitations of traditional imaging methods [7] in resolving the local (and rapidly decaying) *Ca*^2+^ signal triggering the vesicles release. The step-like *Ca*^2+^ uncaging has shown a high sensitivity of the synapse under consideration to a uniform and sustained *Ca*^2+^ elevation in the range of 10 *μ*M (while, otherwise, *Ca*^2+^ concentration must be in the range of 100-300 *μ*M to have an effect). Yet, we needed to produce a spatially uniform rapidly decaying *Ca*^2+^ transient (wave-like calcium uncaging) in order to ascertaining whether very short *Ca*^2+^ elevations, as those following the action potential invasion of the synaptic terminal, suffice to induce vesicle release. Hence, the step-like uncaging has been modeled in order to measure the sensitivity of vesicles to uniform and sustained calcium concentration, whereas the wave-like uncaging has been modeled in order to ascertain the sensitivity of vesicles to short-lived variations of calcium concentration.

**Figure 3:**
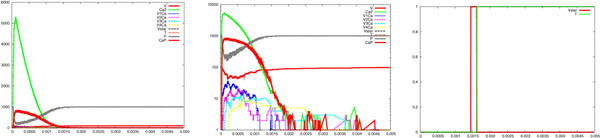
**Wave-like calcium uncaging**. Simulation results showing vesicle release following a wave-like *Ca*^2+^ uncaging. From left to right are reported the time course of the *Ca*^2+^ wave, the logarithmic scale of it, showing the intermediate states of calcium binding, and a focus on vesicle activation (*V star*) and the cumulative amount of vesicle released (*T*). It can be observed that the calcium wave lasts about 1 *ms* with a half width of about 0.5 *ms* and a peak value of about 6000, which corresponds to a peak calcium concentration of about 20 *μ*M. The right side shows the release of one vesicle. The parameters were the same set utilised in the step-like case (see legend of Figure 2). The parameters of the equations describing calcium extrusion were: *c*_1_ = 8 *s*^−l^, *c*_2_ = 25 *s*^−l^ and *c*_3_ = 10000 *s*^−1^.

The experiments and models on *Ca*^2+^ uncaging [7] showed a high sensitivity of vesicle release in response to a uniform elevation of [*Ca*^2+^] in the range 10 *μ*M. It was unclear whether very short [*Ca*^2+^] elevations suffice to induce a release similar to that elicited during an action potential. A recent experimental work [33] has addressed this issue. A spatially uniform and very rapidly decaying [*Ca*^2+^] transient was induced in the presynaptic element of a calyx of Held synapse by *Ca*^2+^ uncaging in the presence of added *Ca*^2+^ buffers. Remarkably, the short-lived elevation of calcium concentration triggers vesicle release. We have introduced in our model a simple mechanism of calcium extrusion utilized in a previously developed model [16], adapting the rate constants to fulfill our needs:

$$Ca_{i}^{2+}+P\begin{array}{c} \overset{c_{1}}{\to }\\ \underset{c_{2}}{\leftarrow } \end{array}CaP\overset{c_{3}}{\to }Ca_{o}^{2+}$$

where $Ca_{o}^{2+}$ is the extruded calcium, *P* is an abstraction of a pumping mechanism, *c*_1_ = 8 *s*^−1^, *c*_2_ = 25 *s*^−1^ and *c*_3_ = 10000 *s*^−1^. Starting with 1000 *P*, we have obtained a simulated calcium wave lasting about 1 *ms* and with a half width 0.5 *ms*, conforming to the experimental requirements [33]. The left side of Figure 3 shows a calcium wave lasting about 1 *ms* with a half width of about 0.5 *ms* and a peak value of about 6000, corresponding to a peak calcium concentration of 20 *μ*M. The right side of the same figure depicts the release of one vesicle. Considering that a whole presynaptic element can be made of about 70 of our simulated clusters, this implies that a single action potential, and accordingly a single calcium wave, is able to release a significant amount of vesicles. This is also along the line of the experimental findings [7,8,33].

### Membrane receptor activity

We have then focused on the postsynaptic side of calyx of Held synapse and we have modeled the part of the postsynaptic membrane functionally related to the presynaptic mechanisms previously described. Our model considers postsynaptic membranes consisting of AMPA receptors and has been derived from a previously developed deterministic model, see [14,34] for details.

The equations of our stochastic model are:

$$C_{0}+T\begin{array}{c} \overset{r_{b}}{\to }\\ \underset{r_{u}^{1}}{\leftarrow } \end{array}C_{1}+T\begin{array}{c} \overset{r_{b}}{\to }\\ \underset{r_{u}^{2}}{\leftarrow } \end{array}C_{2}\;\;\;\;\;\;\;\;\;\;C_{2}\begin{array}{c} \overset{r_{o}^{2}}{\to }\\ \underset{r_{c}^{2}}{\leftarrow } \end{array}O_{2}\;\;\;\;\;\;\;\;C_{2}\begin{array}{c} \overset{r_{o}^{1}}{\to }\\ \underset{r_{c}^{1}}{\leftarrow } \end{array}O_{1}\;\;\;\;\;\;\;\;C_{2}\begin{array}{c} \overset{r_{d}}{\to }\\ \underset{r_{r}}{\leftarrow } \end{array}D$$

Slightly abusing the notation, the symbol *T* represents here the neurotransmitter molecules that bind to the receptor-gated channels, while in the presynaptic model it has been used to represent the whole content released by a single vesicle. Receptor-gated channels *C*_0_ are activated through a process of two reversible *T* binding steps: from *C*_0_ to *C*_1_, intermediate channel states, and then to *C*_2_, the activated channels. Either the activated channels evolve to the states *O*_1_ and *O*_2_, open to the ion flux transmitting the signal (with different kinetics); or the activated channels evolve to the desensitized states *D*, which are not permeable to the ion flux. In the second case, the fraction of channels that open during a synaptic response decreases. All transitions are reversible.

The synaptic response, on the postsynaptic side, is determined by the time course of the rising phase of the synaptic ion current. This is a function of the opening rate of the receptor-gated channels and of the neurotransmitter concentration. We monitor the numbers of open *O*_1_, *O*_2_ and densensitized *D* channels.

It is worth noting that the number of the *T* stochastically assumed to be released in the synaptic cleft has been determined coherently with the amount stochastically produced by a single calcium wave in the presynaptic terminal in our experiments (about 3 to 6 vesicles). Each “presynaptic” *T* corresponds to a wave of neurotransmitter whose duration and amplitude has been calculated on the basis of several hypotheses, below discussed.

The values of the kinetic rates are: *r_b_* = 400 *s*^−1^, $r_{u}^{1}$ = 6 *s*^−1^, $r_{u}^{2}$ = 86000 *s*^−1^, *r_d_* = 900 *s*^−1^, *r_r_* = 64 *s*^−1^, $r_{o}^{1}$ = 100000 *s*^−1^, $r_{o}^{2}$ = 2000 *s*^−1^, $r_{c}^{1}$ = 2000 *s*^−1^, $r_{c}^{2}$ = 250 *s*^−1^. These figures have been determined from the deterministic models in [5,14], accordingly to the transformation already described in the section Step-like calcium uncaging.

In the definition of *r_b_*, the “activation” rate of the receptor, a further difficulty arose: in order to determine the values of the stochastic rate constant, we not only need to estimate the *volume* occupied by the neurotransmitter in the cleft, but also the effective *interaction volume* where the interactions happen against the postsynaptic *surface*. We have computed the *volume* occupied by the neurotransmitters by knowing the number of them contained into a single vesicle (6000-8000) and the value of their concentration when they have spread into the synaptic cleft: 10^−3^*M*. We obtained *volume* = 10^−17^*liter*. Following [13], we estimated the effective *interaction volume* by dividing *volume* by 1/160, obtaining the result of 6 10^−20^*liter*. We adopted this value for the conversion of the deterministic rate constant into the stochastic one, obtaining *r_b_* = 400. Finally, the *interaction volume* determined permits us to estimate the number of neurotransmitters involved: about 50 *T*, as peak value. The value of *C*_0_, which represents the number of membrane channels, has been assumed to be 100 according to known data, see e.g. [5].

**Figure 4:**
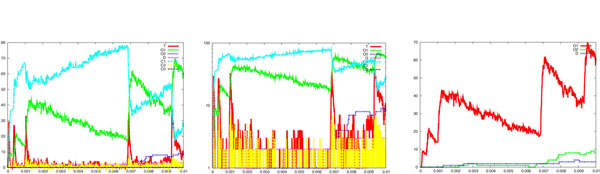
**Membrane receptor activity.** Simulation results showing the postsynaptic membrane receptor activity, which follows the release of neurotransmitters. From left to right are reported the time course of the neurotransmitter *T* and the channel states *C*_0_, *C*_1_, *C*_2_, *O*_1_, *O*_2_ and *D*, the logarithm scale of them and a focus on the time course of *O*_1_, *O*_2_ and *D*. Each presynaptic stochastically generated *T* corresponds to a wave of neurotransmitters whose duration and amplitude have been calculated on the basis of several hypotheses (see text). It can be observed the very short duration of the *T* wave if compared to the slower kinetics of the postsynaptic activated channels. For each *T* wave, the number of open channels is of the order of tens and it is also evident a buildup of the desensitization. The values of the kinetic rates were: $r_{b}^{1}$ = 400, $r_{u}^{1}$ = 1/6, $r_{u}^{2}$ = 86000, *r_d_* = 900, *r_r_* = 64, $r_{o}^{1}$ = 100000, $r_{o}^{2}$ = 2000, $r_{c}^{1}$ = 2000, $r_{c}^{2}$ = 250,*C*_0_ = 100.

We have performed a series of simulations and we report a sample of our results in Figure 4, which consists of three pictures showing the time course of the neurotransmitter *T* and the channel states *O*_1_, *O*_2_, *D*, *C*_1_, *C*_2_, *C*_0_; the logarithmic scale of them; and a focus on the time course of *O*_1_, *O*_2_, *D*. It can be noted the very short duration of the *T* waves and a slower kinetics of the postsynaptic activated channels (compared to the duration of the *T* waves). This corresponds to the prolonged time course of the synaptic currents experimentally observed [34,35]. Moreover, it can be observed that for each *T* wave, i.e. for each vesicle released according to our assumptions, the number of open channels is of the order of tens, fitting with experimental observations [5]. Finally, the buildup of the desensitization is also evident, again coherently with experimental data.

### Complete synapse signal traversal

A comprehensive model of the whole synapse has been devised by building upon the independently developed models of the pre- and post- synaptic processes, which have been considered in isolation in the previous sections. The calcium pulse induces the release of vesicles, represented abstractly in the presynaptic model as the release of a *single* neurotransmitter *T* by each vesicle. In the postsynaptic model, each *T* has been interpreted as a wave of neurotransmitters, with amplitude and duration determined as above. It must be underlined that in the study of synaptic transmission many of the elements we considered are generally neglected, such as the stochasticity of the vesicle release and the calculation of the real amount of neurotransmitter sensed by the postsynaptic receptors.

**Figure 5:**
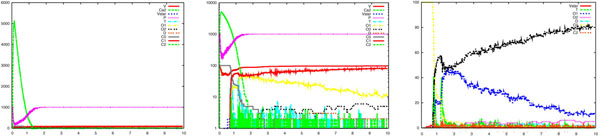
**Complete synapse model: from wave-like calcium uncaging to postsynaptic channel opening**. Simulation results of the synaptic model devised by building upon the independently developed models of the pre- and post- synaptic processes, considered in isolation. From left to right, it can be observed the calcium wave amongst other values, the logarithmic scale of them, which highlights the dynamics of the process: two vesicles are released within 2 *ms* and the corresponding two *T* waves are visible. The right part of the figure focuses on a selection of the variables and the time course of the neurotransmitters *T* is distinguishable. It can be observed that these waves lead to the opening of some receptor channels in a very short time and lasting few *ms* longer. It must be noted that the *T* waves are induced stochastically by the calcium waves.

In Figure 5, we report a sample of results about the simulation of the model regarding a calcium wave with the same mechanisms and coefficients of those in the section about wave-like uncaging. The left part displays the calcium wave, amongst other values. The logarithmic scale of the same picture, in the middle, highlights the dynamics: two vesicles are released within 2ms and the corresponding two waves of neurotransmitters are clearly visible (also clearly distinguishable in the right part, the *T* curve). These waves start processes that lead to the opening of some receptor channels in a very short time and lasting for few milliseconds longer. This is coherent with experimental data, as described in the section about membrane receptors. It is worth noting that the *T* waves we supplied when describing the membrane receptor dynamic in isolation, are now induced stochastically by the calcium wave.

Beyond validating the consistency of the model, some preliminary experiments have also been run about open issues in short-term plasticity (reported in the subsequent section about plasticity). Indeed, the interaction between presynaptic and postsynaptic mechanisms generates short-term plasticity events in a way not yet fully understood [15].

### Parameter sensitivity analysis

We performed a parameter variation study (sensitivity analysis) in the case of step-like and wave-like calcium uncaging and for the postsynaptic terminal. The sensitivity analysis is a standard procedure in modeling and is performed with the aim of evaluating the robustness of the model, i.e. the tolerance of the release process to parameter variation, and to identify, if any, critical parameters.

For the presynaptic terminal, we have run simulations for different values of the number of vesicles (reference value 100, other values: 10, 50, 200 and 500), the number of calcium ions *Ca*^2+^ (reference values 300, 3000 and 6000, other values: 12000, 18000, 24000), the stochastic coefficients *c_on_* (reference value 0.3, other values: 0.1, 0.2, 0.4 and 0.5), *c_coff_* (reference value 9500, other values: 5500, 7500, 11500 and 13500), *b* (reference value 0.25, other values: 0.1, 0.2, 0.3 and 0.4) and γ (reference value 6000, other values: 2000, 4000, 8000 and 10000). One of the results of this analysis is that the forward coefficient *c_on_* seems to have a critical role: higher values of this coefficient correspond to a faster release, in the step-like case, and to a switch from no-release to a consistent release, in the wave-like case. A selection of analysis results is reported below.

**Figure 6:**
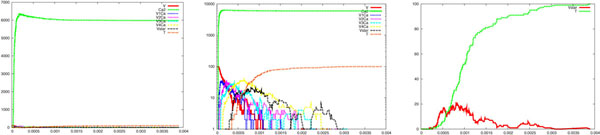
**Parameter variation: step-like case**. Simulation results showing how vesicle release is sensitive to an increase of the parameter *c_on_*, from 0.3 (reference case, see Figure 2) to 0.5, in the case of step-like *Ca*^2+^ uncaging. See legend of Figure 2 for details. It can be observed a high increase of the release rate, when compared to the reference case (Figure 2, right side).

**Figure 7:**
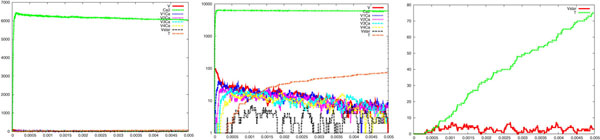
**Parameter variation: step-like case.** Simulation results showing how vesicle release is sensitive to an increase of the parameter *b*, from 0.25 (reference case, see Figure 2) to 0.4, in the case of step-like *Ca*^2+^ uncaging. See legend of Figure 2 for details. It can be observed a lower and more uniform release rate, when compared to the reference case (Figure 2, right side).

For the step-like case, Figures 6 and 7 show runs for *c_on_* = 0.5 and *b* = 0.4, respectively. In Figure 6 we observe a high increase of the release rate, while in Figure 7 we observe a lower and more uniform release rate, both compared to the reference case (Figure 2). A simple variation of these coefficients changes the dynamics of the release processes in an unpredictable manner, producing in one case a variable and decreasing rate of release (see the change in the slope of the curve in between 1 and 1.5 *ms* in Figure 6) and in the other case a stationary rate of release (Figure 7). This kind of experiments are of interest when addressing the problem of the variations in the release rate, one of the still obscure phenomena which have been observed about vesicle release. These variations have been explained by the recruitment of new vesicles within the same active zone or by a different sensitivity to calcium ions of the vesicles belonging to the same cluster. Further investigations with our approach are ongoing about these controversial experimental data [7,8,33].

**Figure 8:**
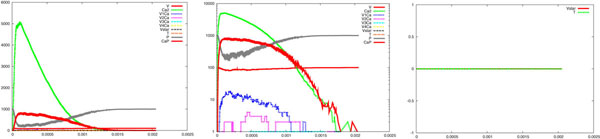
**Parameter variation: wave-like case**. Simulation results showing how vesicle release is sensitive to a decrease of the parameter *c_on_* from 0.3 (reference case, see Figure 3) to 0.1, in the case of wave-like *Ca*^2+^ uncaging. See legend of Figure 3 for details. It can be observed that no release happens (compare to the reference case of Figure 3, right side).

**Figure 9:**
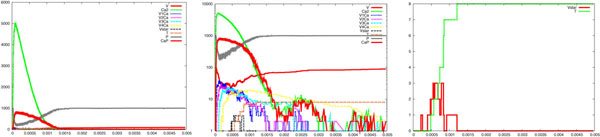
**Parameter variation: wave-like case**. Simulation results showing how vesicle release is sensitive to an increase of the parameter *c_on_* from 0.3 (reference case, see Figure 3) to 0.5, in the case of wave-like *Ca*^2+^ uncaging. See legend of Figure 3 for details. It can be observed a significant increase of the release, up to 8 vesicles in 1 *ms* (compare to the reference case of Figure 3, right side).

For the wave-like case, Figures 8 and 9 show runs for *c_on_* = 0.1 and *c_on_* = 0.5, respectively. It is interesting that in Figure 8 no release appears, whereas in Figure 9 a significant release (up to 8 vesicle in 1 *ms*) can be observed. These two different behaviour are obtained with the same (shape, peak value and duration) calcium wave. Hence the same very short lived calcium wave can be not enough or, on the contrary, even too much for inducing vesicle release. This is certainly a significant finding for the interpretation of recent experimental results, in which most of the attention has been directed, so far, only to the issue of calcium concentration variation [33]. A modulation of this coefficient might be a way for the synapse to store information, increasing or reducing the vesicle release rates (and ultimately it might be relevant for memory and learning).

We have also performed sensitivity analysis of parameters for the postsynaptic model. While the simulated channel dynamics appears overall suitably coherent with the experimental findings, the understanding of the parameter variation results is still under investigation. Amongst the other performed experiments, we report on the variation of two parameters. These two experiments are sufficient in order to illustrate the great variability of the roles of the postsynaptic parameters, which, indeed, are still poorly understood.

**Figure 10:**
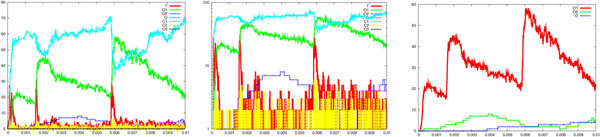
**Parameter variation: membrane receptor activity.** Simulation results showing how postsynaptic membrane receptor activity following neurotransmitter release is sensitive to an increase of the parameter $r_{u}^{1}$ from 6 (reference case, see Figure 4) to 60. This coefficient is relevant for the unbinding rate of the transmitter-channel complex. See legend of Figure 4 for details. The variation of one order of magnitude seems to not significantly alter the kinetics of the main opening channel mechanism, represented by *O*_1_ (compare the rightmost pictures of this figure and Figure 4).

Figure 10 illustrates the simulation results obtained by varying of the coefficient $r_{u}^{1}$ from 6 to 60. This is relevant for the unbinding rate of the transmitter-channel complex. This variation of one order of magnitude does not seem to significantly alter the kinetics of the main opening channel mechanism, represented by *O*_1_ (compare the rightmost pictures of Figure 10 and Figure 4). The slight variations in the kinetics of *O*_2_ and *D* could be explained in terms of stochastic fluctuations.

**Figure 11:**
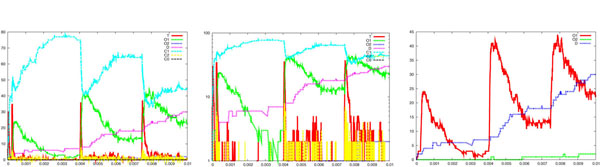
**Parameter variation: membrane receptor activity.** Simulation results showing how postsynaptic membrane receptor activity following neurotransmitter release is sensitive to an increase of the parameter *r_d_* from 900 (reference case, see Figure 4) to 9000. This coefficient represents the rate of desensitization. See legend of Figure 4 for details. In this case, the variation of one order of magnitude of this coefficient is relevant: the time course of *D* is faster and this impact on the peak capability of *O*_1_ (compare the rightmost pictures of this figure and Figure 4).

Figure 11 illustrates the simulation results obtained by varying the coefficient *r_d_* from 900 to 9000. This represents the rate of desensitization. In this case the effects of a variation of the same order of magnitude as before are more marked: the time course of *D* is clearly faster and this impacts on the peak capability of *O*_1_, too.

### Plastic events throughout the complete synapse

**Figure 12:**
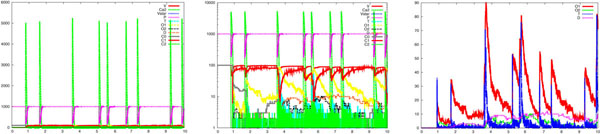
**An *in silico* experiment on the complete synapse model. **Results about an experiment regarding a train of calcium waves at a frequency of about 100 Hz, which mimics action potentials like neural signals. In the left picture calcium waves and other variables are reported. The same data are reported in the center picture in logarithmic scale. This allows the dynamics of the many processes involved to be appreciated, such as the dynamics of the neurotransmitters and the number of open channels. In the right picture a selection of the most significant variables is reported. It can be noted, for instance, that the same amount of neurotransmitter causes different effects as far as the number of opened channels is concerned (compare the ratio between *O*_1_ and *T* in the first two waves with the same ratio in the sixth and the seventh wave) and, above all, a buildup of desensitization is visible starting from the fourth wave (see the increase of value *D* in between the third and fourth wave).

Some experiments addressing plasticity events in the whole synapse have been carried out using the complete synapse model. Besides describing some events of short-term plasticity, our model helps distinguishing the presynaptic and postsynaptic influence in these processes. It is know that in the process of short-term synaptic depression, distinct roles are played by vesicle depletion and postsynaptic receptor desensitization. It has also been shown that desensitization has a role in the synaptic depression only when the frequency of the action potentials is above 10 Hz [15]. Figure 12 shows results about an experiment regarding a train of calcium waves at a frequency of about 100 Hz, which mimics action potentials like neural signals. The left part displays calcium waves, and other values, shown in the central part in logarithmic scale. This makes clear the dynamics of the many processes involved, e.g. that of the neurotransmitter and the number of open channels. The right part shows a selection of the most significant values. Remarkably, a buildup of desensitization is visible starting from the fourth wave (see the increase of value *D* in between the third and fourth wave). Note, also, that the same amount of neurotransmitter causes different effects as far as the number of opened channels is concerned (compare the ratio between *O*1 and *T* in the first two waves with the same ratio in the sixth and the seventh wave). One advantage of our approach is the possibility of quantifying the number of vesicles (recall that each vesicle correspond to a *T* wave with a peak of about 40 neurotransmitters and it is possible to see superpositions of some of them) stochastically associated to each action potential and of measuring at the same time the number of receptor channels open or desensitized. This means that it is possible to distinguish effects due to vesicle depletion, when it builds up, from the effects due to receptor channels.

## Conclusions

Our results are encouraging about the validity of the stochastic approach in studying the synaptic process, which consists of many discrete-like events and involves, for instance, arrays of vesicles and many different molecules. In the process of synaptic transmission, many of these molecules play roles still not fully understood [6]. We have studied parts of the process by adapting data from a number of experimental models. In doing this, we have overcome some limitations of several of these models, e.g. the assumption that concentrations are controlled and homogeneous, so that, for instance, the issue of spatial locality can be not considered. Our discrete approach has instead allowed us to address locality-related issues, like determining the actual interaction surface in the synaptic cleft, according to the quantity of released neurotransmitters and the volume they occupy. This approach appears amenable to also describe other “spatial” aspects of interest.

We built our model in a compositional manner. We first developed a presynaptic model and a postsynaptic model. Once tuned and validated against available experimental data, these models have been integrated to describe the signal traversal throughout the whole synapse. The compositional properties of the chosen approach play a crucial role here: designing and implementing a thin interface was sufficient to obtain the executable model for the whole synapse, with no change in those for the pre- and the post-synaptic processes.

Our model can be further refined along several directions. For instance, the use of a single uniform cluster of releasable vesicles is correct when the process is studied in a short interval of time. When events take place during long-lasting neural activity, more details on vesicle trafficking and cluster compartimentalisation should be introduced. Moreover, the vesicle release is regulated by many intracellular signal pathways, which influence the number and the speed of recruitment of the release-competent vesicles [36].

Embedding these aspects in the model might shed further light on the ways the nervous system processes and stores information. In order to support these, and others, developments the underlying process calculus needs extensions, too. Surely, the problem of expressing locality, already tackled by several calculi, could be valuably addressed within a stochastic viewpoint, together with the mentioned issue of model interfaces.

## Methods

**Figure 13:**
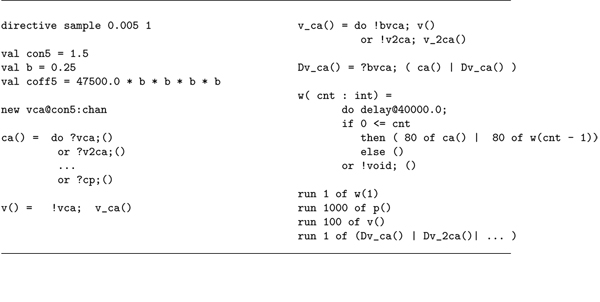
**The calyx of Held SPiM code.** This figure reports a sample of the code implementing the synapse stochastic model. Details are discussed in the section Methods.

In order to provide a grasp of the synapse formal model based on the Pi-calculus, we briefly sketch some of its parts regarding the presynaptic trait. Some excerpts from our model are reported in Figure 13. The language models interaction as pairs of input/output actions over the same communication channel (?c/!c). These atomic actions can be composed in a sequence (!c;?d; !c) or in alternative choices (?c or ?d) so as to form a possibly parametric process (p(…)= …). Processes can run in parallel (p() |q()). Initially, a command sets the duration of the simulation (here 0.005*s*), then some of the stochastic parameters are defined. Communication channels can be (dynamically) created by the new command and have associated a stochastic rate (this and the current quantities of reactants determine the probability of a reaction to “happen” through the channel). In our model one calcium ion, represented by the process ca() without any parameter, can interact with a vesicle v() over the channel vca with rate con5=1.5 (beyond being able to do other things). After this communication, ca() disappears (it becomes the null process ()) and v() becomes v_ca(), which represents the binding of the two. This realises a second order reaction. First order reactions are modeled as interactions with a *single* dummy molecule (so as not to alter stochastic dynamics). For instance, v_ca() can then either accept other calcium bindings, engaging in a second order reaction, or degradate back to an unbound vesicle v() by communicating with the dummy Dv_ca() through bvca, in a first order reaction. Dv_ca() restores ca() and itself.

So far, one can easily realise how the system has been described by specifying simple atomic behaviour, basically corresponding to chemical reactions, and then composing them together. The parametric process w(cnt:int) allows us to suitably modulate the calcium wave. After a stochastic delay, if its integer parameter cnt is positive then it replicates 80 copies of itself, with the parameter decreased, and 80 parallel copies of ca(), otherwise it dies. This realises an exponential growth, which can be controlled in its rapidity and quantity by the delay rate and the parameter. Finally, the initial state can be populated specifying how many molecules of each specie are present (one calcium wave, 1000 pumping molecules, 100 vesicles and the needed dummy molecules). The SPiM simulator produces the dynamics shown in the Figures 2,3,4,5,6,7,8,9,10,11,12.

It is also worth underlining the advantages provided by the compositionality of the approach, well shown by the construction of the complete synapse model. There, two models about different processes (happening in separate volumes), independently developed and tuned, have been connected by suitably mapping boundary processes, i.e. by defining a suitable interface between the models of the pre- and the post-synaptic parts. What was abstracted as the *T* single neurotransmitter released by a vesicle has become in the complete model a suitable wave of release. Actually, the presynaptic t() process now becomes the tt(1) process, which is the wave generator used in the postsynaptic model to spawn the expected amount of neurotransmitters.

Perhaps it could be worth studying whether the concept of interface, well defined in computer science, could be rethought in this context so as to facilitate the modular composition of stochastic models.

## Competing interests

The authors declare that they have no competing interests.

## Authors contributions

Each of the co-authors contributed equally to this project. More precisely, the first and the last author brought their competence in process calculi, and the second and the third their knowledge of neurophysiology, while they carried on jointly the design of the experiment, the modelization of the synapse and the analysis of the results.
